# Conventional clock drawing tests have low to moderate reliability and validity for detecting subtle cognitive impairments in community-dwelling older adults

**DOI:** 10.3389/fnagi.2023.1210585

**Published:** 2023-08-29

**Authors:** Kristen E. Kehl-Floberg, Timothy S. Marks, Dorothy F. Edwards, Gordon M. Giles

**Affiliations:** ^1^Institute for Clinical and Translational Science, University of Wisconsin-Madison, Madison, WI, United States; ^2^Department of Kinesiology-Occupational Therapy, University of Wisconsin-Madison, Madison, WI, United States; ^3^Department of Occupational Therapy, Samuel Merritt University, Oakland, CA, United States

**Keywords:** clock drawing, reliability, validity, cognitive screening, older adults, community sample, mild cognitive impairment

## Abstract

**Background:**

Early identification of subtle cognitive decline in community-dwelling older adults is critical, as mild cognitive impairment contributes to disability and can be a precursor to dementia. The clock drawing test (CDT) is a widely adopted cognitive screening measure for dementia, however, the reliability and validity of paper-and-pencil CDT scoring scales for mild cognitive impairment in community samples of older adults is less well established. We examined the reliability, sensitivity and specificity, and construct validity of two free-drawn clock drawing test scales–the Rouleau System and the Clock Drawing Interpretation Scale (CDIS)–for subtle cognitive decline in community-dwelling older adults.

**Methods:**

We analyzed Rouleau and CDIS scores of 310 community-dwelling older adults who had MoCA scores of 20 or above. For each scale we computed Cronbach’s alpha, receiver operating characteristic curves (ROC) for sensitivity and specificity using the MoCA as the index measure, and item response theory models for difficulty level.

**Results:**

Our sample was 75% female and 85% Caucasian with a mean education of 16 years. The Rouleau scale had excellent interrater reliability (94%), poor internal consistency [0.37 (0.48)], low sensitivity (0.59) and moderate specificity (0.71) at a score of 9. The CDIS scale had good interrater reliability (88%), moderate internal consistency [0.66 (0.09)], moderate sensitivity (0.78) and low specificity (0.45) at a score of 19. In the item response models, both scales’ total scores gave the most information at lower cognitive levels.

**Conclusion:**

In our community-dwelling sample, the CDIS’s psychometric properties were better in most respects than the Rouleau for use as a screening instrument. Both scales provide valuable information to clinicians screening older adults for cognitive change, but should be interpreted in the setting of a global cognitive battery and not as stand-alone instruments.

## 1. Introduction

Mild cognitive impairment (MCI) is estimated to affect about 7% of adults aged 60–65, increasing to 25% by age 80–85 years (Petersen et al., 2018). MCI is defined by the American Psychiatric Association as subtle decline from baseline cognition accompanied by decreased performance of cognitively-complex daily activities (American Psychiatric Association [APA], 2013; Petersen et al., 2018). Evidence guiding clinical screening of cognition during routine health care for healthy older adults is evolving along with recent advances in the understanding of both normal aging and neurocognitive disease (Livingston et al., 2020). Recognition has grown that healthy older adults may benefit from cognitive screening as part of preventative health care (Anderson, 2019) because cognitive impairment contributes to disability (Jekel et al., 2015; Lindbergh et al., 2016) and has been found to progress into dementia in about 15% of cases (Petersen et al., 2018). In clinical practice, accurate early identification of mildly-impaired cognition enables assessment and treatment of causes of reversible cognitive decline (e.g., sleep or mood disorders), pursuit of non-pharmacological interventions to support cognitive aging (Tsolaki et al., 2011), and management of comorbidities that contribute to dementia risk (Livingston et al., 2020). Thus there is a critical need for rapid, reliable, and sensitive objective screening tools that detect subtle cognitive change from baseline (Anderson, 2019).

However, to meet these evolving clinical needs, more evidence is needed on the value of cognitive screening in healthy older adults. The U.S. Preventive Services Task Force rates the evidence for benefit from screening asymptomatic older adults for cognitive decline as insufficient (Lin et al., 2013), and many cognitive screening instruments may not be reliable for this purpose as they were designed to detect dementia, not subtle impairment. The clock drawing test (CDT) is one such classic cognitive screening instrument. The CDT is a paper-and-pencil tool developed for dementia screening, administered by asking the patient to draw an analog clock face with a specific time setting which is then scored by the assessor using a scale of criteria for success. Although developed over one hundred years ago (Hazan et al., 2018), it has recently gained interest for its potential to detect mild impairment (Duro et al., 2019), its neurofunctional correlations with the executive functions required to complete complex daily activities (Royall et al., 1999; Dion et al., 2022), and an adaptation to digital format (Müller et al., 2017; Davoudi et al., 2020; Dion et al., 2020; Yuan et al., 2021).

Clock drawing is of interest for screening healthy older adults based on its plausible neurocognitive relevance to milder impairment (Strauss et al., 2006) such as its reliance on constructional praxis and executive function and their neuroanatomical correlates (Talwar et al., 2019) which are often affected in early dementia and MCI (Mendez et al., 1992; Rouleau et al., 1992, 1996; Chiu et al., 2008; Talwar et al., 2019). A free-drawn electronic CDT using an instrumented pen and electronic tablet-style writing surface has been found to distinguish people with MCI from healthy controls (Yuan et al., 2021), supporting the assumption that the cognitive skills required to draw a clock are relevant to the diagnostic purpose of screening for mild impairment. Compared to this electronic version, paper versions are not as accurate in detecting subtle change (Müller et al., 2017); however, conventional versions are more practical, brief, easy to obtain and administer, familiar [a CDT task is incorporated into several test batteries such as the Mini-Cog (Lam et al., 2011) and the Montreal Cognitive Assessment (Nasreddine et al., 2005)], and still have potential to yield rich clinical information. This makes conventional CDT appealing as a screening instrument for mild impairment in community-dwelling older adults.

Despite its benefits and potential, further examination of conventional CDT’s appropriateness for detection of subtle impairment is needed. First, some of the index measures used in initial validation studies have since been shown to be insufficiently sensitive to MCI in a range of patient populations (Arevalo-Rodriguez et al., 2021), so these psychometric references do not relate to current diagnostic practice or evidence. Second, it is unknown whether the scoring ranges for many versions, scaled to detect potential dementia, can detect small but meaningful decreases from an unimpaired cognitive baseline. Third, although paper-and-pencil CDT’s have been shown to have low sensitivity to MCI (Pinto and Peters, 2009; Duro et al., 2019), only a few recent studies have focused on community-dwelling older adults (one of the most important populations to screen for MCI). Particularly in older studies establishing cut scores, study design precluded conclusions about mild impairment. Finally, we were not able to find previous work that used methods such as item response theory to determine whether these scales’ levels of difficulty are suitable for screening this population. Although item response theory has been employed to evaluate tests that include a clock drawing task [e.g., the Montreal Cognitive Assessment (Luo et al., 2020), the Texas Functional Living Scale (Lowe and Linck, 2021)] its application to stand-alone clock scales has not been reported. Because (a) the CDT was developed primarily to detect dementia, (b) the diagnostic scope of neurocognitive disorder has since expanded to include MCI, (c) gold standard screening tools are now more sensitive to MCI, and (d) more evidence is needed for community-dwelling older adults who are the target population for screening, further study is needed to confirm conventional clock drawing’s psychometric properties for detecting small differences between unimpaired and mildly impaired cognition in community-dwelling older adults.

This study examined the reliability, sensitivity and specificity, and construct validity of two CDT scoring systems, the Rouleau (Rouleau et al., 1992, 1996) and the Clock Drawing Interpretation Scale (CDIS) (Mendez et al., 1992), for identifying cognitive performance consistent with MCI in community-dwelling older adults. We hypothesized that (a) the CDIS would exhibit higher reliability and validity than the Rouleau, and (b) both scales would distinguish people with subtle cognitive deficits from cognitively unimpaired individuals at a score close to the maximum. Additionally, we will examine the construct validity of both scales’ measurement of cognitive ability.

## 2. Materials and methods

### 2.1. Design

This cross-sectional observational study was approved by the University of Wisconsin-Madison Institutional Review Board. All participants provided written informed consent.

### 2.2. Participants

A convenience sample of *N* = 345 community-dwelling older adults was recruited in and around Madison, Wisconsin through flyers posted in community spaces, in-person recruitment at community events, and word-of-mouth. Inclusion criteria were age 55 years and older, living in the community, self-reported independence with daily activities, and ability to read and write in English.

### 2.3. Instruments

To obtain clock drawings, participants were instructed to draw a clock free-hand with the time setting “ten past eleven.” These drawings were scored on the Rouleau and CDIS scales by two licensed and registered occupational therapists (TM, GG) and two occupational therapy graduate students under their supervision. These scores were combined into one data set, and identifiers linking scores with raters were removed.

#### 2.3.1. Criterion measure: the Montreal Cognitive Assessment

The Montreal Cognitive Assessment (MoCA) is a brief screening of cognition (Nasreddine et al., 2005). It is a 10-min, paper-and-pencil screen of global cognition, encompassing multiple neurocognitive domains. It is scored from 0 to 30. For broad use, score ranges are designated at 30–26 for unimpaired cognition, 25–20 for performance consistent with mild cognitive impairment, and 0–19 consistent with dementia (Nasreddine et al., 2005); however, alternative cut scores of 24 (Townley et al., 2019) to 23 (Carson et al., 2018) have been proposed for community-dwelling older adults and other specific populations (Milani et al., 2018; Rossetti et al., 2019; Townley et al., 2019). Compared to the Mini Mental Status Exam (the criterion measure in the original validation papers for these clock scales), the MoCA has superior sensitivity to MCI (90% for MoCA versus 18% for the MMSE) and good positive and negative predictive values (89 and 91%, respectively). As the criterion measure for the present study, the MoCA has the added advantage of a CDT task with the same “face, numbers, hands” structure as the Rouleau and CDIS.

#### 2.3.2. Clock drawing test scales: the Rouleau and the Clock Drawing Interpretation Scale

We selected these scales for several characteristics that maximize their suitability for mild impairment screening. First, they have similar structures; the scoring criteria are all sequentially organized by the clock face, numbers, and hands. The relative contributions of each of these aspects is only partially understood, however, errors in hand placement have been suggested as indication of need for further assessment (Esteban-Santillan et al., 1998). Second, their difficulty is among the highest of the CDT versions because the clocks are drawn free-hand using a three-step command, which has been found to induce more errors than copied versions (Chiu et al., 2008). This suggests a more difficult task, which may be more likely to discriminate between persons with and without mild impairment. Third, they are among the largest and most detailed item banks of all the paper-and-pencil versions, increasing their potential reliability (Bandalos, 2018a). Finally, the clinical utility of this type of scale–using an unadjusted score based on criteria that emphasize qualitative aspects of performance–have been found to have the best clinical utility (Shulman, 2000). See Appendix A for score criteria.

##### 2.3.2.1. Rouleau clock drawing test scale

Rouleau’s scale is a modified version of a scale published by Sunderland et al. (1989); Rouleau et al. (1992). It is scored from 0 (most impaired) to 10 (least impaired), divided into three polytomous items: the clock face (worth 2 points), numbers (4 points), and hands (4 points). To develop the items, Rouleau et al. completed a “qualitative error analysis” of clocks from 50 participants with dementia (25 with AD, 25 with Huntington’s disease), and found six dimensions of errors which they then formalized as items. Rouleau did not report the qualitative methodology for error identification, reliability, or validity of this instrument; it has been found to be sensitive to dementia but less so to MCI (Chiu et al., 2008; Duro et al., 2019).

##### 2.3.2.2. Clock Drawing Interpretation Scale (CDIS)

The Clock Drawing Interpretation Scale (CDIS) is a 20-item rating scale developed from clock drawing errors made by persons with Alzheimer’s disease (Mendez et al., 1992). In a sample of forty-six people with dementia recruited from a memory clinic and twenty-six neurologically-healthy older adult controls (*N* = 72), the CDIS was found to have excellent inter-rater reliability (*r* = 0.95), and internal consistency (*r* = 0.95). Its highest concurrent validity was found for constructional praxis, at a moderate level (*r* = 0.65–0.66). The cut score for dementia was 19. Psychometric properties for individuals with MCI have not been reported for the CDIS.

### 2.4. Statistical analysis

Descriptive statistics were computed and graphically-assessed for distribution. Internal consistency was assessed using Cronbach’s alpha (Cronbach, 1951). Sensitivity and specificity of the two CDT scales to mild impairment were assessed using the receiver operating characteristic area under the curve (ROC AUC) (Zweig and Campbell, 1993) for MoCA cut scores of 26 (Nasreddine et al., 2005) and 23 (Carson et al., 2018). The area under the curve is an estimate of the percentage of times that a true positive is detected (*specificity*) and a false negative is avoided (*sensitivity*), ranging from 0.5 (no better than chance) to 1.0 (accurate 100% of the time). Our two cut scores were chosen to compare the clock’s validity against the original mild impairment cut score of 26 (Nasreddine et al., 2005) versus Carson et al.’s (2018) recommendation for better sensitivity in community samples of 23. Finally, we tested the construct validity of the individual items and the three-aspect structure (face, numbers, hands) using Item Response Theory methods to estimates the scales’ correspondence to cognitive level. For the Rouleau method’s hierarchical polytomous items, we tested the assumptions of dimensionality and ordinal structure using the Graded Response Model (Samejima, 2010), and then tested the amount of information given about cognitive ability using factor analysis with a 3-factor model. For the CDIS’s dichotomous items, we tested the information given about cognitive ability using a 2-parameter model (2PL). An alpha level of 0.05 was used for all statistical tests. Analyses were computed in R version 4.2.2 (R Core Team, 2022) using the psych (Revelle, 2022), ltm (Rizopoulos, 2022), and mirt (Chalmers, 2012) packages.

To confirm scoring fidelity, we tested interrater reliability for a subset of cases. The first author selected a random sample of *n* = 18 participants using a simulation-based random number selection in R software, scored these participants’ drawings on both scales, then computed weighted kappa statistics for each scale comparing their scores to the original scores in the data set. The kappa statistics were 0.94 for the Rouleau and 0.88 for the CDIS, indicating good to excellent interrater reliability.

## 3. Results

### 3.1. Participants

We administered assessments to 345 community-dwelling older adults aged 55 years and older, retaining 310 for this analysis after excluding 35 people with MoCA scores below 20, as individuals with possible dementia are outside this study’s focus on mild impairment; this resulted in a final sample of 310 participants. Our sample was predominantly female (75%) and Caucasian (86%), with a mean age 67 (8.9) years, and a mean of 16 (3.2) years of education. The overall mean score on the MoCA was 25 (2.7), on the Rouleau was 9 (1.5), and on the CDIS was 18 (1.9). The sample’s MoCA scores were about evenly split between unimpaired/impaired at a cut score of 26; a larger proportion were classified as unimpaired with a cut score of 23. Table 1 presents demographics and test scores.

**TABLE 1:** Sample characteristics.

	MoCA cut of 26			MoCA cut of 23		
*N* = 310	MoCA in unimpaired range (26–30) *n* = 146 (47%)	MoCA in mild impairment range (20–25) *n* = 164 (53%)	*Test, p*-value	MoCA in unimpaired range (23–30) *n* = 241 (78%)	MoCA in mild impairment range (20–22) *n* = 69 (22%)	*Test, p*-value
Sex, *n* (%)			*X^2^* = 2.3, NS			*X^2^* = 1.9, NS
Female, 233 (75%)	116 (79%)	117 (71%)		186 (77%)	47 (68%)	
Male, 77 (25%)	30 (21%)	47 (29%)		55 (23%)	22 (32%)	
Race, *n* (%)			NS*			NS*
White, 266 (86%)	132 (91%)	134 (82%)		217 (90%)	49 (71%)	
Black/African American, 33 (10%)	8 (6%)	25 (15%)		17 (7%)	16 (23%)	
Other, 3 (<1%)	5 (3%)	5 (3%)		6 (2%)	4 (6%)	
Ethnicity, *n* (%)			NS*			NS*
Hispanic/Latino, 9 (3%)	5 (3%)	4 (2%)		7 (3%)	2 (3%)	
Not Hispanic/Latino, 298 (96%)	140 (97%)	158 (98%)		232 (97%)	66 (97%)	
Age; mean (SD) 69.85 year (8.9)	67 (6.6)	73 (9.8)	*t* = −6.03 <0.001	69 (8.3)	73 (10)	*t* = 2.79, 0.006
Education; mean (SD) 16 year (3.2)	17 (3.2)	15 (2.9)	*t* = 4.84,<0.001	16 (3.2)	14 (2.5)	*t* = 6.00, <0.001
Instrument total scores; mean (SD)**						
MoCA, 25 (2.7)	28 (1.2)	23 (1.6)	*t* = 28.51, <0.00	26 (2.0)	21 (0.75)	*t* = 31.29, <0.00
Rouleau, 9 (1.5)	9 (1.4)	9 (1.4)	*t* = 3.74,<0.001	9 (1.4)	9 (1.4)	*t* = 3.50, <0.00
CDIS, 18 (1.9)	19 (1.8)	18 (1.9)	*t* = 3.44,<0.001	19 (1.9)	18 (1.8)	*t* = 2.26, <0.00

MoCA, “Montreal Cognitive Assessment”; CDT, “clock drawing test”; CDIS, “Clock Drawing Interpretation Scale”; ADCS, “Alzheimer’s Disease Cooperative Study–Activities of Daily Living Scale.”

* Insufficient group representation to run *X*^2^ as either full or collapsed factor variable.

** Assessment statistic results are rounded to whole numbers to fit their scoring conventions.

### 3.2. Reliability

Cronbach’s alpha (α) was computed to estimate the internal consistency of each clock drawing scale. The Rouleau coefficient α was 0.367 (SD 0.48, 95% CI 0.23, 0.48), indicating poor internal consistency, and the CDIS coefficient α was 0.665 (SD 0.09, 95% CI 0.61, 0.72) indicating moderate internal consistency. Table 2 summarizes these reliability estimates. See Supplementary Tables 1, 2 for item statistics.

**TABLE 2:** Summary of test statistics.

	MoCA cut score of 26				MoCA cut score of 23			
	**Rouleau**		**CDIS**		**Rouleau**		**CDIS**	
	**Test statistic (sd)**	**CI**	**Test statistic (sd)**	**CI**	**Test statistic (sd)**	**CI**	**Test statistic (sd)**	**CI**
Cronbach’s α	0.37 (0.48)	(0.23, 0.48)	0.66 (0.09)	(0.61, 0.72)				
**ROC**								
AUC	0.68	(0.59, 0.71)	0.65	(0.59, 0.71)	0.66	(0.59, 0.73)	0.63	(0.56, 0.69)
Sensitivity*	0.59		0.78		0.66		0.83	
Specificity*	0.71		0.45		0.61		0.37	

CDIS, “Clock Drawing Interpretation Scale”; ROC, “receiver operating characteristic”; AUC, “Area under the curve.” MoCA cut score is not used to compute Cronbach’s α.

* At a score of 9 on the Rouleau and 19 on the CDIS.

### 3.3. Sensitivity and specificity

**FIGURE 1:**
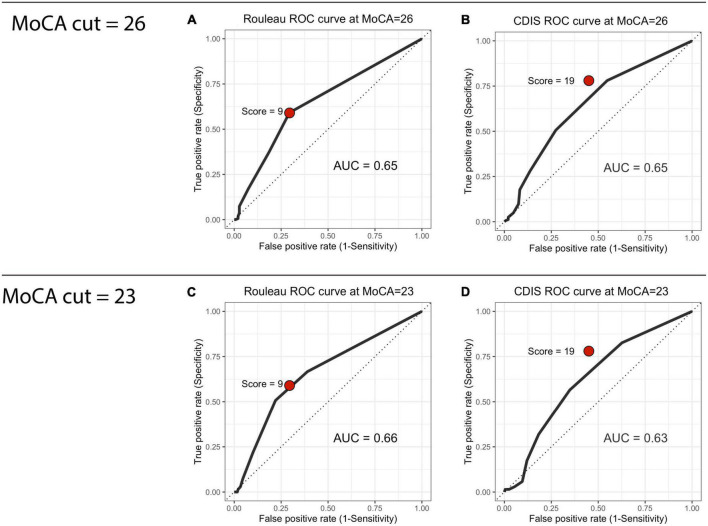
Receiver operating characteristic curves. **(A)** Rouleau ROC curve at MoCA = 26, **(B)** CDIS ROC curve at MoCA = 26, **(C)** Rouleau ROC curve at MoCA = 23, **(D)** CDIS ROC curve at MoCA = 23.

We computed ROC curves for both clock drawing scales to establish criterion (or “cut” scores) using the MoCA as the index measure. We computed each scale’s area under the curve (AUC), sensitivity (the proportion of scores below the CDT’s cut score to our two cut scores on the MoCA) and specificity (the proportion of scores above the cut score on the CDT to the scores above the given cut score). At a MoCA cut of 26, the AUC values were 0.65 for both the Rouleau (0.5945–0.7055) and the CDIS (0.5897–0.7087). At a MoCA cut of 23, these were 0.66 (0.5905–0.7305) for the Rouleau and 0.63 (0.5590–0.6985) for the CDIS. Curves are presented in Figure 1. CDT scale cut scores were determined with a threshold analysis showing the optimal sensitivity and specificity balance. At a MoCA cut of 26, a Rouleau score of 9 (out of 10) had sensitivity of 0.59 and specificity of 0.70, and a CDIS cut score of 19 (out of 20) had sensitivity of 0.78 and specificity of 0.45. At a MoCA cut of 23, a Rouleau score of 9 had sensitivity of 0.66 and specificity of 0.61, and a CDIS cut score of 19 had sensitivity of 0.82 and specificity of 0.37. Table 2 summarizes these statistics.

### 3.4. Construct validity

#### 3.4.1. Rouleau

**FIGURE 2:**
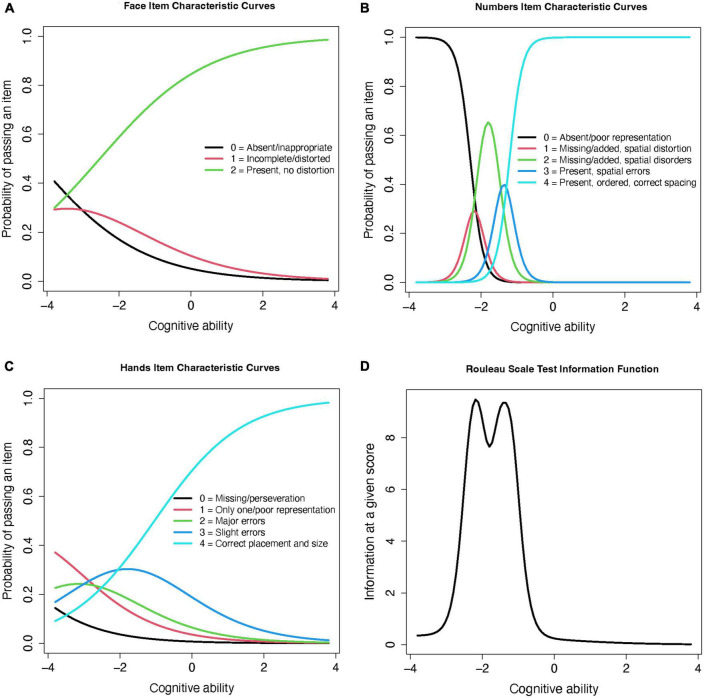
Rouleau item and test information curves from the graded response model. **(A)** Face Item Characteristic Curves **(B)** Numbers Item Characteristic Curves **(C)** Hands Item Characteristic Curves **(D)** Rouleau Scale Test Information Function.

Because this scale uses an ordinal item structure, the graded response model (GRM) was used (Samejima, 2010). The GRM estimates the item-wise probabilities of satisfying each criterion (or response level) within each item (progressing from low to high cognitive ability), and a value of the “threshold” between levels, which estimates the item’s distinction between each criterion (an important characteristic of an ordinal item). The item information curves (Figures 2A-C) show that the probability of passing more difficult items (*y* axis) increases with cognitive ability (*x* axis), but that the level of ability required to pass them is lower than the average cognitive level of our participants. The clearest differentiation between performance on each criterion is in the “hands” item, with distinct differences in the cognitive abilities contained within each score. This suggests that this item clearly discriminates between high, middle, and low cognitive levels, and is therefore a true ordinal item. By contrast, the “face” and “numbers” items’ middle scores are nearly contained within the lowest and highest score levels, suggesting that they are less discriminative and have very close thresholds–this suggests that, in practice, they are more likely to work as binomial (impaired/unimpaired) than ordinal (step-wise with multiple levels) items. The test information curve (Figure 2D) also shows that this scale yields the most information for participants with lower cognitive performance. The Rouleau items contributed insufficient degrees of freedom to assess goodness-of-fit using root mean square error of approximation (RMSEA) (Cai and Hansen, 2013); the marginal residual were heterogeneous, ranging 5.00 (“face” and “hands”) to 21.74 (“numbers” and “hands”).

**FIGURE 3:**
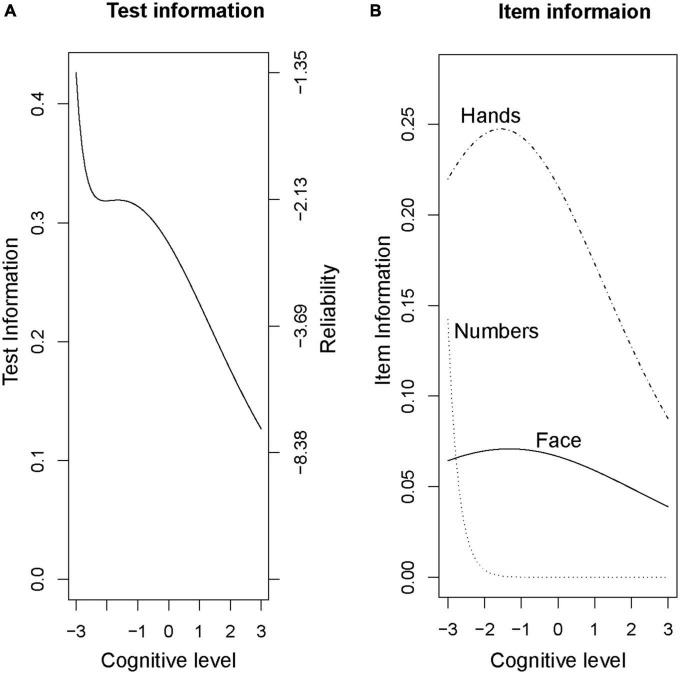
Rouleau factor analysis model, Total and Item Curves. **(A)** Test information **(B)** Item information.

To allow comparisons between the Rouleau and CDIS scales, we then ran a factor analysis on the Rouleau scores using a 3-factor item loading model. This model placed the Rouleau scale’s difficulty below the average cognitive level in this sample (−8.28 to −0.54). The “face” aspect was the least discriminating (0.38) and “numbers” was most discriminating (3.58). Figure 3 shows changes in difficulty across cognitive levels (x axis) for the total score (Figure 3A) and each item (Figure 3B). Note that the curves for “numbers” are shifted to the left, suggesting that this component is easier to score a point on than “hands.” The curve for “face” is virtually flat, suggesting it does not give much discriminating information. The overall leftward shift of the curves shows that this scale provides more information at a lower cognitive level, which is consistent with the graded response modeling above.

#### 3.4.2. CDIS

**FIGURE 4:**
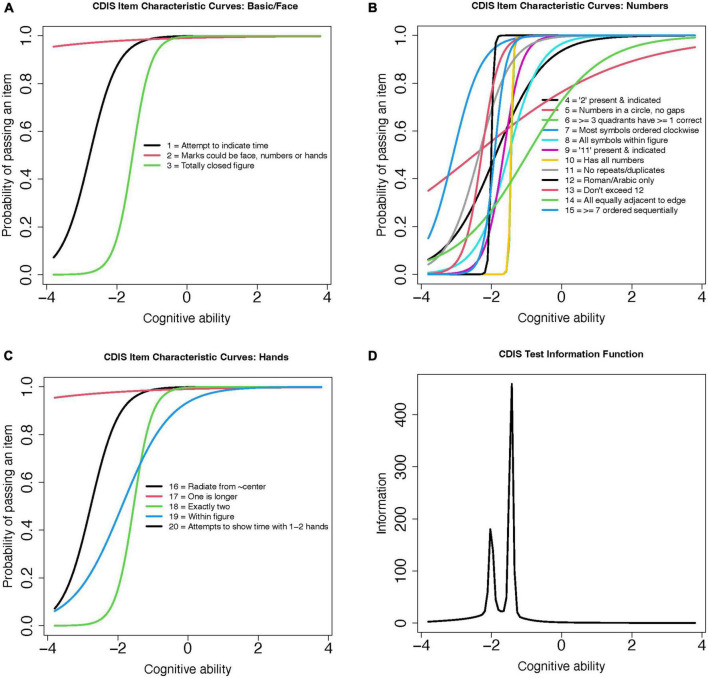
CDIS 2-Parameter model (2PL) test information curves. **(A)** CDIS Item Characteristic Curves: Basic/Face **(B)** CDIS Item Characteristic Curves: Numbers **(C)** CDIS Item Characteristic Curves: Hands **(D)** CDIS Test Information Function.

This scale’s binomial (0 or 1 point) item structure was assessed using a 2-parameter model (2PL). The 2PL model includes parameter values for item discrimination (α-parameter) and difficulty (β-parameter) to for each CDIS item. The coefficient values for these estimates are plotted for the items grouped by aspect (Figures 4A-C) and total score (Figure 4D) as a test information curve. As was the case for the Rouleau, this curve shifts to the left; this shows that the CDIS scale yields the most information for participants with poorer cognitive performance, but with more restricted range of abilities compared to the Rouleau scale. Model fit was estimated using RMSEA; a value of 0.09 indicated fair to poor model fit (Boateng et al., 2018).

## 4. Discussion

In this community-dwelling sample of older adults, we found that the CDIS had moderate reliability (though superior to the Rouleau scale), and that both scoring systems were moderately sensitive to MoCA scores consistent with mild cognitive impairment. Our item response theory models showed that both scales provided very limited information about small shifts toward impaired cognition, and that the “hands” items appeared to be the most discriminating. These findings are consistent with several prior findings on the Rouleau scale in particular (Chiu et al., 2008; Pinto and Peters, 2009; Talwar et al., 2019). Thus, our hypotheses were partially supported; the CDIS scale was more reliable (but not more sensitive) than the Rouleau scale, and a score of only one unsatisfied criterion had the highest sensitivity to mild impairment. Based on these results, we found insufficient evidence to recommend either scale be used as a stand-alone screening instrument in this population.

Of the two scales, the CDIS showed higher internal consistency, which we expected since reliability tends to increase as the number of items in a scale increases. One possible contribution to our low reliability is item composition. Quantitative ratings of a free-drawn object require some amount of subjective interpretation, so each item must be written for narrow interpretation with unmixed criteria for success (Bandalos, 2018b). Best practices include giving one criterion for success per item, avoiding qualifiers, and using single clauses (Bandalos, 2018b). Several of these recommendations are violated in these two scales. For instance, the Rouleau method uses qualifiers - such as “most,” “minor,” and “slight”–in 8 out of 10 items, and the CDIS in 3 out of 20. These qualifiers are adjectives expressing relative degree, but neither scale provides metrics to guide clinicians in placing performance between degrees. The influence of wording on reliability could be explored in the future by comparing item-total correlations and item statistics to identify the items with the lowest correlation to the total score, then dropping the low-correlated items and re-analyzing the scales.

Another consideration is our reliability estimator’s underlying assumptions and limitations. We chose Cronbach’s alpha to allow comparisons with prior work (Duro et al., 2019), however, there is debate as to whether this statistic is an appropriate estimator of internal consistency for this type of scale (Cho, 2021; Sijtsma and Pfadt, 2021). First, use of the alpha statistic is based on the assumption that the items of the scale are *unidimensional*, that is, they measure the same construct. Although theses scales are screening measures for dementia (one construct), they require many overlapping abilities such as visuospatial, executive function, motor and perceptual integration, and a cultural context for chronological measurement of time (multiple constructs)–thus they may not measure dementia *per se*, but rather multiple skills as proxies of dementia. Second, the *error variance of all items should be uncorrelated*–that is, items should vary independently of one another (Trizano-Hermosilla and Alvarado, 2016). In the assumptions of classical true score theory that underlies alpha, error originates unpredictably from a random process, so item variances should not influence one another. This is unlikely to apply to these scales because elements of a drawing rely on previous elements, for instance the clock’s hand placement relies on the clock face being symmetrical and the numbers evenly spaced. Third and finally, the scale’s items should be *essentially tau-equivalent*, meaning they may differ in mean and variances but must have similar covariances; if this assumption is violated then alpha is likely to underestimate the scale’s true reliability (Bandalos, 2018a). On the other hand, Cronbach’s alpha has also been found to mask multidimensionality of test items, and slightly overestimate ordinal scales such as the Rouleau scale (Vaske et al., 2017). We retain Cronbach’s alpha for this study as a lower-bound estimate of reliability, and acknowledge that other methods, such as a stratified alpha (estimations for face, numbers, and hands singly), the greatest lower bound, or expanded factor analytic methods could provide more accurate estimates for this type of scale in a larger sample.

Our criterion validity results were generally consistent with other recent work. At the MoCA cut score of 26, the sensitivity and specificity of these clock scales were moderate, and the AUC point estimates were nearly identical to that of another recent ROC analysis of the Rouleau scale (Duro et al., 2019). Lowering the criterion score to 23 had a negligible effect on AUC, but shifted both clock scales’ sensitivity 5–6 points higher, specificity 8–10 points lower, and confidence intervals several points wider, suggesting lower precision. At either cut, the highest sensitivities were lower than the 90% (Nunnally, 1978) to 95% (Kaplan and Sarcuzzo, 2001) recommended for a clinical screening tool, found at a score of only one incorrect item (9/10 on the Rouleau, 19/20 on the CDIS).

There is evidence that the MoCA score of 26 is overly sensitive, resulting in too many false positives (Carson et al., 2018; Townley et al., 2019). With deference to the inconvenience and cost this can cause, we argue that in high-functioning community-dwelling adults it is preferable for a non-invasive and brief screening tool to err on the side of greater sensitivity with the potential for false positives, rather than greater specificity with the potential to overlook cases. The MoCA is a screening measure to detect potential impairment, not to diagnose (Dautzenberg et al., 2020), and administering clinicians will also consider trends, subjective report, and patient concerns in balance with scores. Compared to the studies recommending lower cut scores (Carson et al., 2018; Milani et al., 2018; Townley et al., 2019), our sample had higher mean education in both impaired and unimpaired groups regardless of classification score, and very low racial and ethnic heterogeneity. Our ROC results at a cut score of 23 may be more valid for community samples with low mean education (Carson et al., 2018) and people from underrepresented racial groups (Milani et al., 2018; Rossetti et al., 2019); however, we could not verify this with our sample.

Our application of item response theory models is the first use of these methods with these two scales. Our results help clarify the relationship between ability and test performance, a key aspect of the test’s appropriateness for subtle cognitive decline. In our sample, these scales had levels of difficulty that were more appropriate to rating performance by a person with a lower level of cognition than the early or subtle decline we hope to capture in community-dwelling older adults; in other words, the scoring criteria on these scales appear to be too easy for this population. We found a ceiling effect that begins to detect change well below an unimpaired level, in turn driving the scales downward in cognitive ability and resulting in restriction of range that masks performances with subtle impairments. We note that both scales’ test information curves are bimodal with a leftward shift (right skew), suggesting that they are very informative for a narrow range of ability at a low cognitive level, and not informative for very small decreases from baseline. This is consistent with prior study on their psychometric properties for detecting dementia (Mendez et al., 1992; Chiu et al., 2008) and was also consistent with our and others’ ROC analyses (Duro et al., 2019). Hence, many of the items in these scales contributed little to no information toward characterizing subtle change in our sample. A caveat is that the GRM and 2PL models had poor indicators of fit–the model “expects” a response pattern that differed from that of our sample. We suspect two sources of this high error approximation; the scales may contain poorly-performing items that introduce noise into the model, and, the task’s step-by-step nature may constitute multidimensional latent constructs contributing to each item. Both of these hypotheses could be explored using exploratory factor analysis or other latent trait modeling in a future study.

It is also notable to consider the change in diagnostic definition and practice since these scales were developed. These scales’ purpose was to detect impairment within a binomial “dementia vs. no dementia” construct of cognitive function, which contrasts with our current understanding of neurocognitive decline as a continuum. For instance, the original validation paper for the CDIS compared healthy older adults to persons with dementia but excluded those with “questionable” cognitive impairment, establishing just one cut score for dementia rather than a range of scores across the cognitive spectrum. Similarly, the scoring criteria for the Rouleau scale (Rouleau et al., 1992, 1996) were developed according to errors made by people with dementia, and did not characterize a range of unimpaired performance as a reference. At that time, a range of cognitive function was not necessarily considered clinically useful, but this is no longer the case.

Based on our findings, we suggest that the task of drawing a clock is not “too easy” for a person with subtle cognitive deficits to execute without errors, but rather that dementia-validated paper scales used to score their execution may be too lenient. If the scales’ sensitivity and specificity to MCI could be improved, they would meet established criteria for good clinical screening tools (Edwards et al., 2019). Future studies could attempt to improve these scales by repeating testing and analysis after removing items with low item-total correlations and RMSEA, conducting expanded factor analyses, and using alternative reliability estimators.

### 4.1. Limitations

Our study has several limitations. First, our sample is not representative of the population of normally-aging community-dwelling older adults. We had three female participants for every male, and an insufficient number of participants who were Black, Hispanic, or other racial or ethnic identities to compute statistics on these sub-groups. Although our racial and ethnic demographic imbalance isn’t a barrier to comparing these results with the original validation studies for these scales (neither reported these variables), it is a barrier to implementing these findings in broader clinical practice because it does not reflect the proportions of these individuals in the population of people who develop dementia, nor does it inform us on bias in these instruments (Chin et al., 2011; Weuve et al., 2018) particularly given our index measure’s different score distributions in some populations (O’Caoimh et al., 2016; Milani et al., 2018; Rossetti et al., 2019). A second limitation is the lack of cognitive diagnoses for our sample. We used the MoCA (a screening measure with a disputed cut score) to test the sensitivity of the two CDT scales (another screening measure with no established cut score) without scores from a diagnostic criterion measure. Thus, our analyses are limited to observing groups with scores *consistent with*, but not definitively diagnostic of, unimpaired versus mildly impaired cognition. Finally, our design precludes determination of predictive validity because we used a cross-sectional design without a repeating measure.

## 5. Conclusion

Although the task of free-drawing a clock has been found to represent cognitive abilities affected by MCI (Royall et al., 1999; Yuan et al., 2021) our analysis of the CDIS and Rouleau clock drawing scales found that they did not reliably identify persons scoring in the mildly impaired range on the MoCA. We recommend use of these scales in combination with other screening tools primarily as an opportunity to observe visuospatial and executive abilities, with awareness of a ceiling effect in the interpretation of their numeric score.

Cognitive changes are often subtle and granular; even if diagnosis is categorical, small changes are both clinically meaningful for care-planning and functionally meaningful for patients. Assessment tools must reflect small differences in function to be useful for screening community-dwelling older adults. In our community-dwelling sample, the Rouleau and CDIS clock drawing scales did not reflect these small differences.

## Data availability statement

The raw data supporting the conclusions of this article will be made available by the authors, without undue reservation.

## Ethics statement

The studies involving human participants were reviewed and approved by the University of Wisconsin-Madison Health Sciences Institutional Review Board. The patients/participants provided their written informed consent to participate in this study.

## Author contributions

KK-F, TM, GG, and DE: concept, data collection, and design. KK-F: statistical analysis and writing. TM, GG, and DE: editing. All authors contributed to the manuscript revision, and approved the final submission.

## Appendix

Appendix A Rouleau and CDIS scale score rubrics.

**Table:** Rouleau system.

Participant Code		
**1. Integrity of the clock face (maximum; 2 points)**		
	**2: Present without gross distortion**	
	**1: Incomplete or some distortion**	
	**0: Absent or totally inappropriate**	
**2. Presence and sequencing of the numbers (maximum: 4 points)**		
	**4: All present in the right order and at most minimal error in the spatial arrangement**	
	**3: All present but errors in spatial arrangements**	
	**2: Numbers missing or added but no gross distortions of the remaining numbers; numbers placed in clockwise direction; numbers all present, but gross disorders of spatial layout (i.e., hemineglect, numbers outside the clock)**	
	**1: Missing or added numbers and gross spatial distortion**	
	**0: Absence or poor representation of numbers**	
**3. Presence and placement of hands (maximum: 4 points)**		
	**4: Hands are in the correct position and the size difference is respected**	
	**3: Slight errors in the placement of the hands or no representation of size difference between the hands**	
	**2: Major errors in the placement of the hands (significantly out of course including 10 to 11)**	
	**1: Only one hand or poor representation of two hands**	
	**0: No hands or perseveration on hands**	
	**Total**	

**Table:** Clock Drawing Interpretation Scale (CDIS).

Participant Code		
	Criteria	Point
**1**	**There is an attempt to indicate a time in any way**	
**2**	**All marks or items can be classified as either part of a closure figure, a hand, or a symbol for clock numbers.**	
**3**	**There is a totally closed figure without gaps (closure figure)**	
**Score only if symbols for clock numbers arc present**		
**4**	**A “2” is present and is pointed out *in* some way for the time**	
**5**	**Most symbols are distributed as a circle without major saps.**	
**6**	**Three or more clock quadrants have one or more appropriate numbers: 12–3, 3–6, 6–9, 9–12 per respective clockwise quadrant.**	
**7**	**Most symbols are ordered in a clockwise or rightward direction.**	
**8**	**All symbols are totally within a closure figure**	
**9**	**An “11” is present and is pointed out in some way for the time.**	
**10**	**All numbers 1–12 are indicated.**	
**11**	**There are no repeated or duplicated symbols**	
**12**	**There are no substitutions for Arabic or Roman numerals.**	
**13**	**The numbers do not go beyond the number 12**	
**14**	**All symbols lie about equally adjacent to a closure figure edge,**	
**15**	**Seven or more of the same symbol type are ordered sequentially.**	
**Score Only if One or More Hands Are Present**		
**16**	**All hands radiate from the direction of a closure figure center.**	
**17**	**One hand is visibly longer than another hand**	
**18**	**There are exactly two distinct and separable hands.**	
**19**	**Ail hands are totally within a closure figure.**	
**20**	**There is an attempt to indicate a time with one or more hands.**	
	**Total (out of 20)**	
