# Supplementary material for: Conventional clock drawing tests have low to moderate reliability and validity for detecting subtle cognitive impairments in community-dwelling older adults
Source: Front Aging Neurosci. 2023 Aug 29;15:1210585. doi: 10.3389/fnagi.2023.1210585 (PMC10495769; doi:10.3389/fnagi.2023.1210585)
Supplement: Supplementary file 1 [file Table_1.DOCX]

Supplementary Material

Conventional Clock Drawing tests have low to moderate reliability and validity for detecting subtle cognitive impairments in community-dwelling older adults

Kristen Kehl-Floberg*^1^, Timothy S. Marks^2^†, Dorothy F. Edwards^1,2^ , Gordon M. Giles^3^

*** Correspondence:** Corresponding Author: kekehl@wisc.edu

# Supplementary Data

Supplementary Material should be uploaded separately on submission. Please include any supplementary data, figures and/or tables.

Supplementary material is not typeset so please ensure that all information is clearly presented, the appropriate caption is included in the file and not in the manuscript, and that the style conforms to the rest of the article.

--NONE--

# Supplementary Figures and Tables

## Supplementary Tables

**2.1.1 Supplementary Table 1. Rouleau Reliability item statistics**

|  | **Item statistics** | | | | | | **Alpha if item dropped** | | | | | | | |
| --- | --- | --- | --- | --- | --- | --- | --- | --- | --- | --- | --- | --- | --- | --- |
|  | **raw.r** | **std.r** | **r.cor** | **r.drop** | **mean** | **sd** | **Raw alpha** | **Std alpha** | **G6(smc)** | **Average r** | **S/N** | **alpha se** | **Var r** | **Med r** |
| Rouleau Face | 0.478 | 0.595 | 0.183 | 0.109 | 1.765 | 0.551 | 0.438 | 0.447 | 0.288 | 0.288 | 0.810 | 0.062 | *NA* | 0.288 |
| Rouleau Numbers | 0.702 | 0.704 | 0.460 | 0.293 | 3.768 | 0.700 | 0.120 | 0.133 | 0.071 | 0.071 | 0.154 | 0.090 | *NA* | 0.071 |
| Rouleau Hands | 0.780 | 0.685 | 0.418 | 0.257 | 3.490 | 0.888 | 0.192 | 0.197 | 0.110 | 0.110 | 0.246 | 0.089 | *NA* | 0.110 |

**2.1.2 Supplementary Table 2. CDIS reliability item statistics**

|  | **Item statistics** | | | | | | **Alpha if item dropped** | | | | | | | |
| --- | --- | --- | --- | --- | --- | --- | --- | --- | --- | --- | --- | --- | --- | --- |
|  | **raw.r** | **std.r** | **r.cor** | **r.drop** | **mean** | **sd** | **Raw alpha** | **Std alpha** | **G6(smc)** | **Avg r** | **S/N** | **alpha se** | **Var r** | **Med r** |
| **CDIS_C1** | **0.340** | **0.450** | **0.455** | **0.302** | **0.994** | **0.080** | **0.658** | **0.735** | **0.836** | **0.127** | **2.771** | **0.027** | **0.037** | **0.065** |
| **CDIS_C2** | **0.162** | **0.265** | **0.179** | **0.110** | **0.990** | **0.098** | **0.664** | **0.750** | **0.860** | **0.136** | **2.998** | **0.026** | **0.040** | **0.066** |
| **CDIS_C3** | **0.544** | **0.415** | **0.426** | **0.449** | **0.945** | **0.228** | **0.632** | **0.738** | **0.836** | **0.129** | **2.814** | **0.029** | **0.036** | **0.069** |
| **CDIS_N4** | **0.450** | **0.502** | **0.451** | **0.309** | **0.900** | **0.300** | **0.645** | **0.730** | **0.848** | **0.125** | **2.709** | **0.028** | **0.041** | **0.040** |
| **CDIS_N5** | **0.353** | **0.171** | **0.069** | **0.133** | **0.761** | **0.427** | **0.680** | **0.757** | **0.863** | **0.141** | **3.115** | **0.025** | **0.040** | **0.074** |
| **CDIS_N6** | **0.415** | **0.605** | **0.634** | **0.346** | **0.977** | **0.149** | **0.649** | **0.721** | **0.823** | **0.120** | **2.586** | **0.027** | **0.035** | **0.064** |
| **CDIS_N7** | **0.103** | **0.205** | **0.164** | **0.073** | **0.997** | **0.057** | **0.665** | **0.754** | **0.851** | **0.139** | **3.073** | **0.026** | **0.040** | **0.073** |
| **CDIS_N8** | **0.519** | **0.402** | **0.364** | **0.389** | **0.903** | **0.296** | **0.634** | **0.739** | **0.850** | **0.130** | **2.830** | **0.029** | **0.038** | **0.066** |
| **CDIS_N9** | **0.492** | **0.693** | **0.704** | **0.404** | **0.958** | **0.201** | **0.639** | **0.713** | **0.831** | **0.115** | **2.481** | **0.028** | **0.037** | **0.040** |
| **CDIS_N10** | **0.355** | **0.573** | **0.600** | **0.289** | **0.981** | **0.138** | **0.653** | **0.724** | **0.826** | **0.121** | **2.624** | **0.027** | **0.033** | **0.065** |
| **CDIS_N11** | **0.311** | **0.400** | **0.337** | **0.237** | **0.977** | **0.149** | **0.656** | **0.739** | **0.852** | **0.130** | **2.832** | **0.027** | **0.040** | **0.064** |
| **CDIS_N12** | **0.303** | **0.517** | **0.512** | **0.254** | **0.990** | **0.098** | **0.658** | **0.729** | **0.840** | **0.124** | **2.690** | **0.027** | **0.034** | **0.066** |
| **CDIS_N13** | **0.168** | **0.364** | **0.330** | **0.126** | **0.994** | **0.080** | **0.663** | **0.742** | **0.848** | **0.131** | **2.876** | **0.026** | **0.038** | **0.069** |
| **CDIS_N14** | **0.508** | **0.317** | **0.243** | **0.294** | **0.710** | **0.455** | **0.653** | **0.746** | **0.856** | **0.134** | **2.934** | **0.027** | **0.040** | **0.065** |
| **CDIS_N15** | **0.345** | **0.524** | **0.500** | **0.290** | **0.987** | **0.113** | **0.655** | **0.728** | **0.843** | **0.124** | **2.683** | **0.027** | **0.037** | **0.065** |
| **CDIS_H16** | **0.562** | **0.402** | **0.336** | **0.363** | **0.719** | **0.450** | **0.639** | **0.739** | **0.853** | **0.130** | **2.829** | **0.029** | **0.041** | **0.040** |
| **CDIS_H17** | **0.357** | **0.240** | **0.157** | **0.136** | **0.758** | **0.429** | **0.680** | **0.752** | **0.859** | **0.137** | **3.029** | **0.025** | **0.041** | **0.065** |
| **CDIS_H18** | **0.377** | **0.338** | **0.284** | **0.263** | **0.942** | **0.234** | **0.651** | **0.744** | **0.851** | **0.133** | **2.908** | **0.027** | **0.040** | **0.065** |
| **CDIS_H19** | **0.636** | **0.537** | **0.558** | **0.538** | **0.923** | **0.268** | **0.617** | **0.727** | **0.831** | **0.123** | **2.666** | **0.030** | **0.037** | **0.059** |
| **CDIS_H20** | **0.268** | **0.388** | **0.376** | **0.218** | **0.990** | **0.098** | **0.659** | **0.740** | **0.844** | **0.130** | **2.846** | **0.027** | **0.038** | **0.065** |
